# The Gut Microbiota of Broilers Reared with and without Antibiotic Treatment

**DOI:** 10.3390/microorganisms11040876

**Published:** 2023-03-29

**Authors:** Genevieve Greene, Leonard Koolman, Paul Whyte, Catherine Burgess, Declan Bolton

**Affiliations:** 1 Teagasc Food Research Centre, Ashtown, D15 DY05 Dublin, Ireland; 2 School of Veterinary Medicine, University College Dublin, Belfield, D04 W6F6 Dublin, Ireland

**Keywords:** microbiota, broilers, antibiotics, gastrointestinal tract

## Abstract

The aim of this study was to examine the microbiota in broilers reared with and without antibiotics and to investigate differences between the upper, middle and lower sections of the gastrointestinal tract (GIT). One of two commercial flocks was treated with an antibiotic (T) (20 mg trimethoprim and 100 mg sulfamethoxazole per ml in the drinking water for 3 days) and the other was left untreated (UT). The GIT contents of 51 treated and untreated birds were aseptically removed from the upper (U), middle (M) and lower (L) sections. These were pooled in triplicate (n = 17 per section per flock), the DNA extracted and purified, 16S amplicon metagenomic sequencing performed and the resultant data analysed using a range of bioinformatics software. There were significant differences in the microbiota of the upper, middle and lower GIT, and treatment with the antibiotic significantly affected the microbiota in each of these sections. This study provides new data on broiler GIT microbiota and suggests that GIT location is a more important determinant of the constituent bacterial flora rather than the use or otherwise of antimicrobial treatments, at least when applied early in the production cycle.

## 1. Introduction

Antibiotics are used in broiler husbandry to prevent and treat disease [1] and up until 2006 were also used to increase feed efficiency in the European Union [2]. In 2017, antibiotic use for growth promotion was also banned in the United States of America, but this practice is still used in other countries [1].

Previous studies have shown that antibiotic treatment causes significant changes in the broiler gastrointestinal tract (GIT) microbiota and may cause dysbiosis and gut development disorders that negatively impact on broiler physiology and metabolic performance [3]. The immune system in birds treated early in the growth cycle is also adversely affected, resulting in lower concentrations of macrophages in intestinal mucosal tissue, which in turn influences the microbiota throughout the rearing period [4].

The broiler microbiota is also affected by several other factors, including, but not limited to, age, diet/feed type, breed, gender, hygiene, house conditions, litter type and maternal factors as well as probiotics, prebiotics, phytobiotics and phages [5]. Moreover, the microbiota of young broilers is highly variable, and some studies suggest that a relatively stable microbiota is not reached until 20 weeks of age [6], while the bacterial communities of each section of the broiler GIT are also different [7]. The major phyla found in the broiler GIT include Firmicutes, Bacteroidetes and Proteobacteria and the major genera include *Lactobacillus*, *Enterococcus*, *Bacteroides* and *Corynebacterium* [8].

Feed ingested by broilers initially passes into the oesophagus (which contains glands that produce mucus to facilitate passage) and on into the crop where it is temporarily stored before passage into the proventriculus. Gastric juices, hydrochloric acid and digestive enzymes in the proventriculus start the digestion process before it passes to the gizzard. In the gizzard, the feed is masticated by continuous contractions in the presence of pepsin and other proteolytic enzymes [9]. In the small intestine (the duodenum, the jejunum and the ileum), the digestion of the proteins is completed, and nutrients are absorbed through the microvilli. This process requires amylases, trypsin, lipases and carboxypeptidases from the pancreas and bile from the liver via the gallbladder to break down fats. Undigested materials such as non-starch polysaccharides then pass into the large intestine and then into the caeca. Any remaining undigested feed is then fermented with waste materials passing through the colon and out through the cloaca (Rebollar Serrano and Serrano, 2002). Poultry production is one of the fastest growing industries in the world [5,10]. In Ireland, approximately two million broilers are produced across three poultry processing plants each week [11]. The quantities of antimicrobials used in the Irish poultry production sector have not been publicly disclosed, but a recent report stated that since 2017 the only antimicrobial classes used in the poultry industry were penicillin (amoxicillin) and potentiated sulphonamides (sulfamethoxazole, sulfadiazine and trimethoprim) [12]. The administration of antibiotics to broilers requires a prescription from a veterinarian, and approximately half of all treatments take place in the first week of life [12].

The aim of this study was to examine the microbiota in broilers reared with and without antibiotics and investigate differences between the different sections of the GIT, including the upper (including the crop, the proventriculus and the gizzard), middle (including the duodenum, the jejunum and the ileum) and lower (including the large intestine, the caeca and the cloaca) sections of the GIT in both sets of birds.

## 2. Materials and Methods

### 2.1. Description of Samples Used in Study

All broilers (*Gallus gallus domesticus* (Ross breed)) used in this study were raised on a commercial broiler farm that had 2 broiler houses of approximately 30,000 birds each, in county Monaghan, Ireland. The farm had strict biosecurity measures and the 2 flocks used for this study were raised under normal commercial production conditions including feeding regime. One flock was treated with antibiotics, while the other was left untreated. Broiler GIT samples were obtained from the treated (Metaxol^®^, a commercial antibiotic containing 20 mg trimethoprim and 100 mg sulfamethoxazole per ml in the drinking water, at 5 days of age for 3 days) (n = 51) and untreated flock (n = 51), reared in adjacent houses on the same farm at the same time and administered the same feed. The birds were harvested and slaughtered at 32 (untreated) and 33 days (treated). Following evisceration, the GIT were randomly selected from each flock, aseptically packaged and transported at 4℃ to our research facility where they were processed the same day.

### 2.2. Sample Processing

Each GIT was aseptically (in a laminar flow cabinet) divided using a sterile scalpel into upper, middle and lower sections. The crop, the proventriculus and the gizzard were considered to be the upper section, the duodenum, the jejunum and the ileum were the middle section and the large intestine, the caeca and the cloaca made up the lower section. The contents of each GIT section were then aseptically removed to ensure no cross-contamination between the outside and contents of the GIT, pooled in triplicate and stored at −70 °C prior to DNA extraction. This resulted in 17 samples each of treated upper (TU), middle (TM) and lower (TL) as well as the untreated upper (UTU), untreated middle (UTM) and untreated lower (UTL) GIT. DNA was extracted using the Qiagen DNeasy PowerSoil Pro kit (Qiagen, Manchester, UK) according to the manufacturer’s instructions. Following DNA extraction, the quality and concentration of DNA was measured using the NanoDrop spectrophotometer (NanoDrop 1000, ThermoFisher Scientific, Dublin, Ireland).

### 2.3. 16S Amplicon Metagenomic Sequencing of Samples

The DNA was diluted, using sterile water, to a target final concentration of 1 ng/µL. 16S rRNA genes specific to the 16S V3/V4 region were amplified using the 515F-806R primer set [13]. Barcode sequences were attached to the PCR product and the PCR was performed using Phusion^®^ High-Fidelity PCR Master Mix (New England Biolabs, Ipswich, MA, USA). Equal volumes of the resultant PCR product and 1X loading buffer containing SYBR green were combined, visualised on a 2% agarose gel, and samples with a bright band at the 400–450 bp marker were excised using the Qiagen Gel Extraction Kit (Qiagen, Hilden, Germany) for further analysis. DNA libraries were prepared using the NEBNext^®^ Ultra^TM^ DNA Library Prep Kit (New England Biolabs, Ipswich, MA, USA) for Illumina, quantified via Qubit and qPCR. Libraries were analysed on the Illumina NovaSeq 6000 platform (Novogene Bioinformatics Technology Co., Ltd., Beijing, China).

### 2.4. Statistical Analysis

#### 2.4.1. Processing of Sequence Data

Barcodes incorporated into the amplicon were used to assign the paired end reads to each sample. Barcode and primer sequences were then removed and the trimmed paired end reads were merged using FLASH (V1.2.7) [14]. The Qiime (V 1.7.0) quality control process was used to perform quality filtering of these raw tags yielding high quality clean tags [15,16]. Chimeric sequences were detected and removed using the UCHIME algorithm, with the SILVA (release 138) database as a reference, thus producing the effective tags [17,18].

#### 2.4.2. OTU Cluster and Taxonomic Annotation

Sequence analysis was performed on all effective tags using Uparse software (V7.0.1090) [19]. Sequences with ≥97% similarity were assigned to the same Operational Taxonomic Unit (OTU) and a representative sequence for each OTU screened for further annotation. Representative sequences were screened via Qiime (V 1.7.0) using the Mothur pipeline and the SILVA SSU rRNA database to provide species annotation at each taxonomic rank [20,21,22]. MUSCLE (V 3.8.31) was utilised to provide the phylogenetic relationship of all OTUs [23]. Data were normalised and alpha and beta diversity analyses were performed on these normalised data.

#### 2.4.3. Alpha Diversity, Beta Diversity and LEfSe Analysis

Alpha diversity, measuring the complexity and diversity of samples, was analysed with three different alpha diversity indices: namely ACE, Chao 1 and Shannon. Qiime (V 1.7.0) was used to calculate each index and R software (V 2.15.3) was used to visualise the results. Beta diversity, measuring the differences in species complexity of samples, was analysed on both weighted and unweighted UniFrac using Qiime software (V 1.7.0). Cluster analysis was performed using principal coordinate analysis (PCA). FactoMineR and ggplot2 packages in R software (version 2.15.3) were used to reduce the dimensions of the original variables.

Principal coordinate analysis (PCoA) was used to both visualise complex multidimensional data and obtain principal coordinates. A distance matrix of weighted and unweighted UniFrac data was transformed into a new set of orthogonal axes where the maximum factor was demonstrated by first principal coordinate, and the second maximum factor by the second principal coordinate, etc. PCoA analysis was displayed via WGCNA, stat and ggplot2 packages in R software (V 2.15.3). Linear discriminant analysis effect size (LefSe) analysis was performed via LefSe software [24]. The *p*-value was calculated with a permutation test [25]. *t*-test and drawing were conducted using R software (V 2.15.3).

## 3. Results

### 3.1. OTU Identification and Taxonomic Annotation

**Figure 1:**
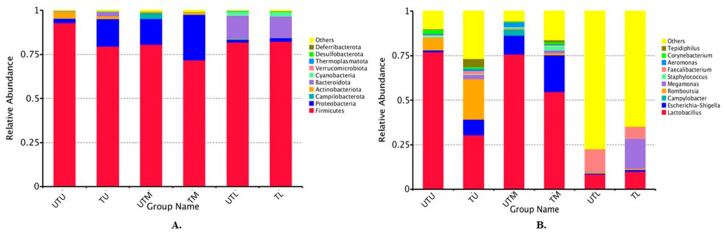
Box charts depicting the top 10 phyla (**A**) and genera (**B**) in both treated (T) and untreated (UT) groups in each section of the GIT (upper (U), middle (M), lower (L)).

A total of 102 samples (17 pooled samples from each GIT section from treated and untreated flocks) were sequenced and each sample had an average of 108,727 effective tags, with each tag having an average length of 420 bp per sequence. The top 10 phyla and genera in the samples are shown in Figure 1. The relative abundance of the different phyla was as follows: TU: Firmicutes (80%), Proteobacteria (16%), Bacteroidetes (3%) and Actinobacteria (1%); TM: Firmicutes (72%), Proteobacteria (26%) and Actinobacteria (1%); TL: Firmicutes (82%), Bacteroidota (12%), Cyanobacteria (3%) and Proteobacteria (2%); UTU: Firmicutes (93%), Actinobacteria (4%), Proteobacteria (2%) and Bacteroidota (1%); UTM: Firmicutes (81%), Proteobacteria (15%), Campliobacterota (3%) and Actinobacteria (1%); and UTL: Firmicutes (82%), Bacteroidota (13%), Cyanobacteria (2%) and Proteobacteria (1%) (Figure 1A).

The top 10 genera in each sample type are shown in Figure 1B. The abundance of the different genera was as follows: TU: *Lactobacillus* (30%), “others” (27%, discussed in further detail below), *Romboutsia* (23%), *Escherichia–Shigella* (9%), *Tepidiphilus* (5%), *Megamonas* (3%), *Faecalibacterium* (2%), *Aeromonas* (1%) and *Corynebacterium* (1%); TM: *Lactobacillus* (55%), *Escherichia–Shigella* (21%), “others” (16%), *Megamonas* (2%), *Staphylococcus* (2%), *Romboutsia* (1%), *Faecalibacterium* (1%), *Corynebacterium* (1%) and *Tepidiphilus* (1%); TL: “others” (65%), *Megamonas* (17%), *Lactobacillus* (10%), *Faecalibacterium* (7%), *Escherichia–Shigella* (1%) and *Romboutsia* (1%); UTU: *Lactobacillus* (77%), “others” (10%), *Romboutsia* (7%), *Corynebacterium* (2%), *Escherichia–Shigella* (1%), *Staphylococcus* (1%), *Aeromonas* (1%) and *Tepidiphilus* (1%); UTM: *Lactobacillus* (76%), *Escherichia–Shigella* (11%), “others” (6%), *Campylobacter* (3%), *Aeromonas* (2%) and *Staphylococcus* (1%); and UTL: “others” (77%), *Faecalibacterium* (12%), *Lactobacillus* (8%) and *Romboutsia* (1%).

**Figure 2:**
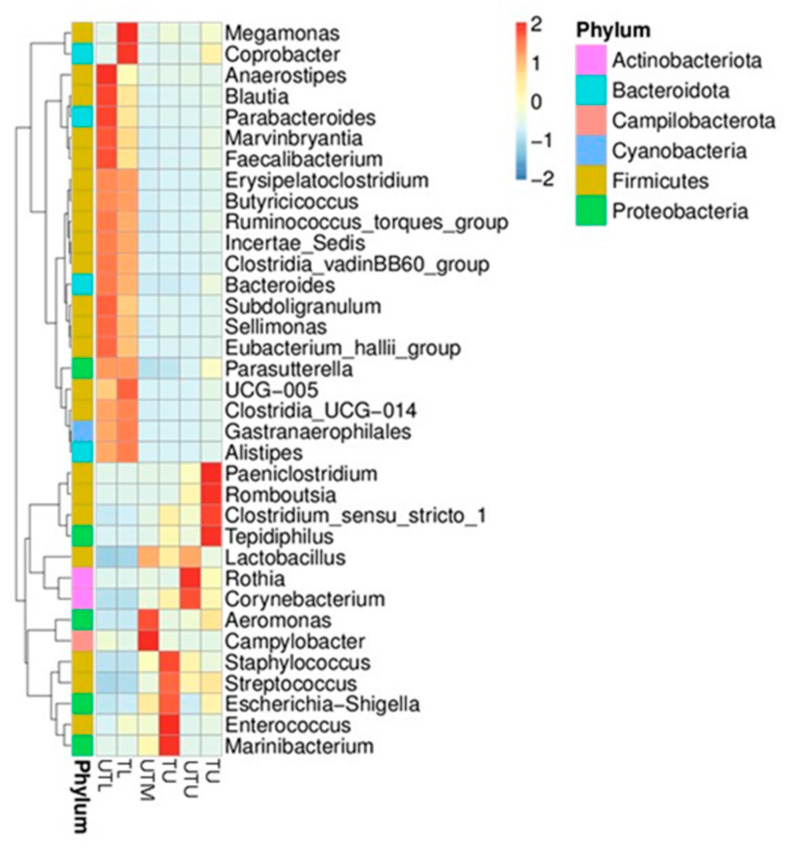
A heat map showing the 35 top genera in the upper (U), middle (M) and lower (L) sections of the GIT of both flocks (treated (T) and untreated (UT)).

Figure 2 depicts a heatmap of the relative abundance top 35 genera in each sample type. *Paeniclostridium*, *Romboutsia*, *Clostridium sensu stricto 1* and *Tedipiphilus* were found to have a higher relative abundance in TU samples; *Staphylococcus*, *Streptococcus*, *Escherichia–Shigella*, *Enterococcus* and *Marinibacterium* had a higher relative abundance in the TM; *Megamonas* and *Coprobacter* had a higher relative abundance in the TL samples; *Rothia* and *Corynebacterium* in the UTU; *Aeromonas* and *Campylobacter* in the UTM, while *Anaerostipes, Blautia*, *Parabacteroides*, *Marvinbryantia* and *Faecalibacterium* were higher in the UTL section. In each GIT section, shifts in the dominant species can be seen between untreated and treated flocks, although less so in the lower GIT.

### 3.2. Alpha Diversity Analysis

**Figure 3:**
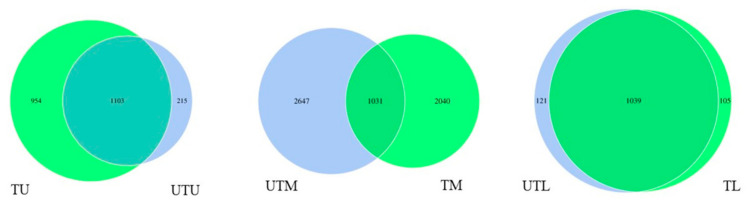
Venn diagrams illustrating the shared and unshared OTUs between each section of the GIT in both flocks. Each circle within the Venn diagram represents a single sample group where U, M and L stands for upper, middle and lower GIT, respectively, and T and UT stand for treated and untreated flocks, respectively.

Venn diagrams were created to illustrate the shared and unshared OTUs between all sections of the GIT in both the treated and untreated flocks (Figure 3). Within the upper GIT, there was a total of 1103 shared OTUs, while there were 954 and 215 unshared OTUs in the treated and untreated flocks, respectively. The middle GIT of both flocks had a total of 1031 shared OTUs, while there were 2040 and 2647 unshared OTUs in the treated and untreated flocks, respectively. Finally, the lower GIT had a total of 1039 shared OTUs, while the treated and untreated flocks had totals of 105 and 121 unshared OTUs, respectively.

**Figure 4:**
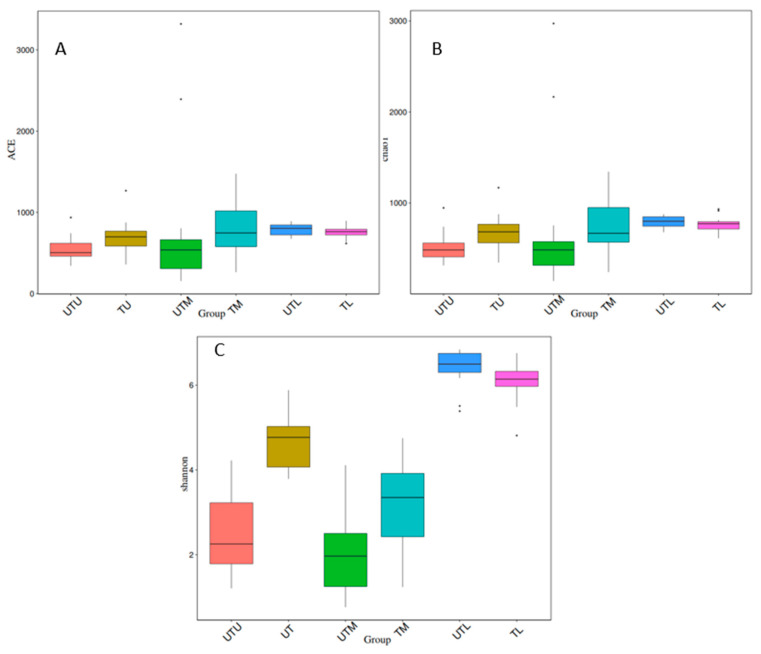
Boxplots depicting three alpha diversity indices. (**A**) Depicts the ACE index, (**B**) depicts the Chao1 index and (**C**) depicts the Shannon index.

Ace, Chao1 and Shannon indices were used to analyse alpha diversity (the diversity within each sample type), and boxplots depicting the results can be seen in Figure 4. The Tukey test and the Wilcox test were applied to determine if the relationship between any two sets of samples was statistically significant, and the results are provided in Table 1. When treated and untreated sections of the GIT were compared (UTU v TU, UTM v TM, UTL v TL), and each section of the GIT was compared to each other (UTU v UTM, UTM v UTL, UTU v UTL, TU v TM, TM v TL and TU v TL), differences in the diversity within the different sample types were statistically significant for at least one diversity index, except for the UTU samples when compared to the UTM samples.

**Figure 5:**
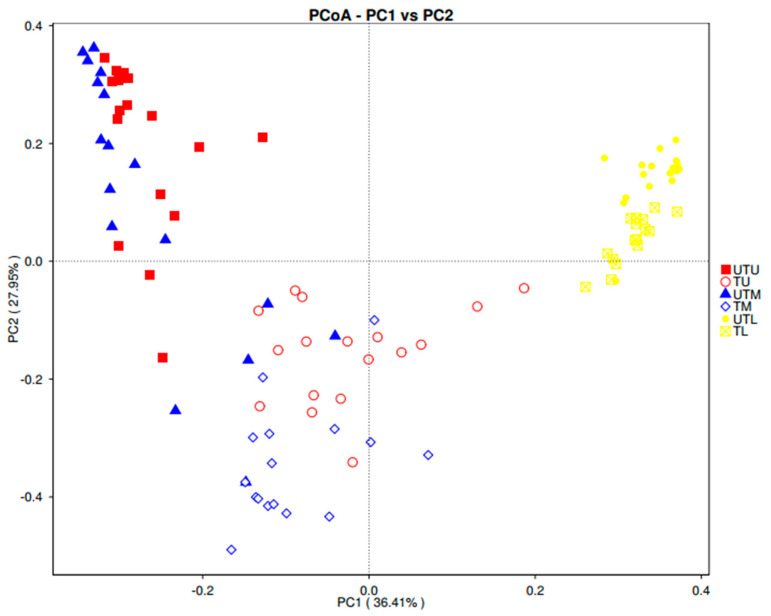
Weighted UniFrac PCoA illustrating the level of similarity between samples.

A weighted UniFrac PCoA plot was used to measure the beta diversity (the number of taxonomic units that are not the same in two different sample types) (Figure 5).

Four distinct clusters were obtained: [1] TL; [2] UTL; [3] TU and TM; and [4] UTU and UTM samples. The close proximity of the lower GIT samples suggests that many of the taxonomic units were the same in these sample types regardless of the treatment status of the birds. The overlapping of the TU and TM data points, distinct from the UTU and UTM cluster, suggests some similarity between the taxonomic units in the upper and middle GIT with greater dissimilarity between treated and untreated samples in these regions.

**Figure 6:**
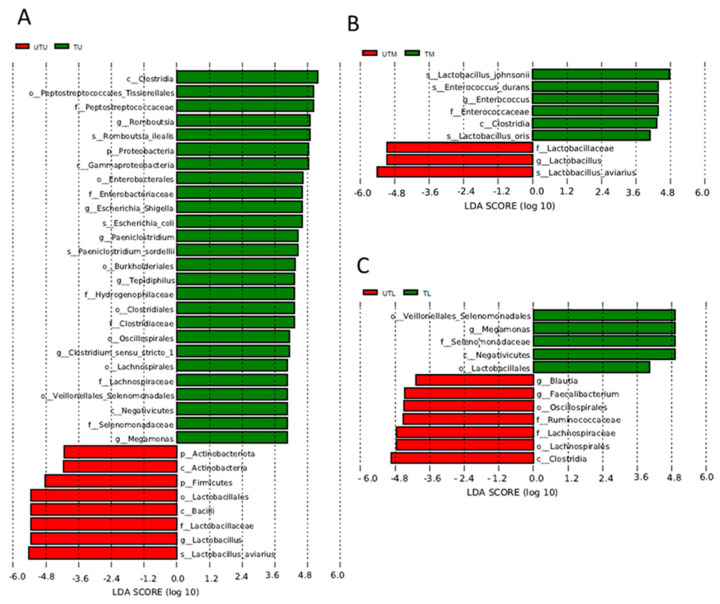
Linear discriminant analysis effect size (LEfSe) illustrating the OTUs responsible for differentiating sample groups from one another. (**A**) UTU vs. TU, (**B**) UTM vs. TM, (**C**) UTL vs. TL.

Linear discriminant analysis effect size (LEfSe) analysis identified the OTUs most likely to account for the differences between the sample types (what distinguishes one sample type from another) (Figure 6). UTU and TU samples were differentiated by the presence of Clostridia, *Tissierellales* (Peptostreptococcales), *Peptostreptococcaceae*, *Romboutsia*, *Romboutsia illealis*, Proteobacteria, Gammaproteobacteria, Enterobacterales, *Enterobacteriaceae*, *Paeniclostridium*, *Paeniclostridium sordelli*, Burkholderales, *Tepidiphilus*, *Hydrogenophillaceae*, Clostridiales, *Clostridiaceae*, *Oscillospiraceae*, *Clostridium sensu stricto* 1, Lachnospirales, *Lachnospiraceae*, Veillonellales/Selenomonadales, Negativicutes, *Selenomonadaceae* and *Megamonas* in the former, and Actinobacteriota, Actinobacteria, Firmicutes, *Lactobacillus*, Bacilli, *Lactobacillaceae*, *Lactobacillus* and *Lactobacillus aviarius* in the latter. UTM and TM samples were differentiated by the presence of *Lactobacillus johnsonii*, *Enterococcus durans*, *Entercoccus*, Clostridia and *Lactobacillus oris* in the untreated samples, and *Lactobacillaceae*, *Lactobacillus* and *Lactobacillus avian* in the treated samples. Finally, the UTL samples had Veillonellales/Selenomonadales, *Megamonas*, *Selenomonadaceae*, Negativicutes and *Lactobacillus*, which were absent in the TL samples, but these had *Blautia*, Faecalibacterium, Oscillospirales, *Ruminococcaceae*, *Lachnospiraceae*, Lachnospirales and Clostridia, which were not detected in the former.

The OTUs that were exclusive to a given sample type included OTUs related to *Tssierellales* (Peptostreptococcales), *Peptostreptococcaceae*, *Romboutsia*, *Romboutsia illealis*, *Paeniclostridium*, *Paeniclostridium sordellii*, Burkholderales, *Tepidiphilus* and *Clostridium perfringens* in the TU samples; Enterobacterales, *Enterobacteriaceae*, *Escherichia–Shigella*, *Escherichia coli*, *Lactobacillus johnsonii*, *Enterococcus*, *Enterococcaceae*, *Enterococcus durans* and *Lactobacillus oris* in the TM samples; *Oscillospiraceae*, *Rikenallaceae*, *Alistipes*, Clostridia UCG 014, Vampirivibronia, Gastranaerophilales and Cyanobacteria in the TL section; Lactobacillales, Bacilli, *Lactobacillaceae*, *Lactobacillus aviarius*, Corynebacteriales, *Corynebacteriaceae* and *Corynebacterium* in the UTU; Aeromondales, *Aeromonadaceae* and *Aeromonas* in the UTM; and Clostridia, *Lachnospiraceae*, Oscillpspirales, *Ruminococcaceae*, Bacteroidales, Bactroidota, Faecalibacterium, Bacteroides, *Bacteroidaceae*, *Bacteroides dorei*, Clostridia vadinBB60 group, *Blautia*, *Ruminococccus torques* group, Subdoligranulum, Parabacteroides, *Tannerellaceae*, *Parabacteroides distasonis* and Erysipelotrichales in the UTL samples.

## 4. Discussion

Firmicutes was the predominant phylum in the broiler GIT (regardless of sample type), with a relative abundance of approximately 72% to 93%, while the other phyla included Proteobacteria (1% to 26%), Bacteroidota (1% to 12%), Actinobacteria (1% to 4%), Campliobacterota (3%) and Cyanobacteria (2% to 3%). Previous studies have also reported Firmicutes as the most abundant phylum in the broiler GIT followed by Proteobacteria and Bacteroidetes [7,8].

There were significant differences in the microbiota detected in the different sections of the GIT. As the different organs along the GIT perform different functions in digestion and nutrient absorption, they create different ecological niches [7]; thus, despite being interconnected, these differences in taxonomic composition are to be expected and have been reported previously [5,6].

Analysis of the diversity suggested that there was significantly lower diversity in the lower as compared to the other sections of the GIT. Stanley et al. [7] also reported significantly less diversity in the lower GIT samples, but other studies have reported the opposite and attributed the higher diversity to the presence of the ceca (Clavijo and Flórez, 2018). These authors hypothesised that the ceca promotes microbial growth and diversity because it is the site of greatest water availability and the undigested feed materials are retained for 12 to 20 h allowing for fermentation of any carbohydrates present [5].

*Lactobacillus* was the predominant genus in the upper and middle GIT samples with relative abundances of 30% (TU), 55% (TM), 77% (UTU) and 76% (UTM) as compared to 10% and 8% in the lower GIT treated and untreated samples, respectively. Clavijo and Flórez [5] also reported relatively high concentrations of *Lactobacillus* in the upper GIT where it has a role in starch digestion and lactate fermentation. *Escherichia–Shigella* was also among the predominant genera with a higher relative abundance in the upper and middle GIT sections (9%, 21%, 1% and 11% in TU, TM, UTU and UTM samples, respectively) as compared to 1% and 0% in the corresponding lower GIT samples. Wei et al. [26] also reported that *Escherichia–Shigella* was a predominant genus in the upper and middle broiler GIT. In contrast, Clavijo and Flórez [5] did not detect these bacteria in the broiler GIT, regardless of location. Moreover, they reported that the crop and gizzard in the upper GIT host several species of the *Clostridiaceae* family, while the small intestine (middle section of the GIT) also contains various species of *Clostridiaceae* and *Enterococcus*. However, these bacteria were not present in the broilers we tested. Thus, while the predominant phyla may be constant (Firmicutes, Proteobacteria and Bacteroidetes), the specific families and genera within these phyla may differ considerably as they are influenced by a range of factors such as feed formulation, environment, sex of the birds, individual genetics, husbandry practices, etc. [5,7].

The application of antimicrobials may also affect the gut microbiota. In our study, while the antibiotic treatment significantly affected the diversity of the microbiota, the effects observed between treated and untreated samples were not as pronounced as those observed between the different sections of the GIT, implying that section of the GIT has a greater effect on the microbiota than antibiotic treatment in the early stages of the production cycle itself. Although data on the effect of antibiotics on the broiler GIT microbiota are scarce, both Elokil et al. [27] and Videnska et al. [28] reported a significant reduction in diversity with 55% to 95% of OTUs disappearing from the treated birds within 48 h in the latter study. However, the same authors also reported the restoration of microbiota complexity after antibiotic withdrawal, which may explain our observations given there were 24–25 days between treatment and sampling.

Other interesting observations included the presence of both helpful and harmful bacteria in the middle GIT samples. *Streptococcus*, for example, found in the treated birds, is a lactic acid producing bacteria, and was previously used as a probiotic in broilers [4] while species such as *Streptococcus faecalis* enhance the systemic antibody response [29]. Human pathogens such as *Aeromonas* and *Campylobacter* were detected in the untreated samples. *Aeromonas* are typically associated with aquatic environments but are also part of the normal microbial flora of both aquatic and terrestrial animals [30,31]. Over the past two decades, several *Aeromonas* species have emerged as human pathogens causing diseases such as gastroenteritis, wound infection and bacteraemia in immunocompromised individuals [32]. *Aeromonas* infection of poultry has been reported in many parts of the world with deleterious effects, and its occurrence in broilers may suggest poultry is a source of infection for humans [32]. *Campylobacter* is of particular interest as it is the most common cause of human bacterial gastroenteritis in the world [33]. There are conflicting reports on whether or not these bacteria also cause disease in the broilers [34,35]. Regardless, it was expected that these bacteria would be found in the lower and not the middle GIT samples, as the caecum is the main site of carriage in broilers [36].

## 5. Conclusions

This study provides new data on the microbiota in the different (upper, middle and lower) sections of the broiler GIT in both treated and antibiotic-free flocks and suggests that both the location within the GIT and antibiotic treatment are important determinants of the constituent bacterial flora even when applied early in the production cycle.

## Figures and Tables

**Table 1:** Statistical significance of ACE, Chao1 and Shannon Indices, which were tested via the Tukey and Wilcox tests.

Samples/*p*-Value	ACE		Chao1		Shannon	
	Tukey	Wilcox	Tukey	Wilcox	Tukey	Wilcox
**UTU v TU**	0.79264	0.0074 ^1^	0.65193	0.0045 ^1^	0 ^1^	0 ^1^
**UTM v TM**	0.98912	0.0011 ^1^	0.98309	0.002 ^1^	0 ^1^	0 ^1^
**UTL v TL**	0.99994	0.5193	0.99987	0.4221	0.76	0.0266 ^1^
**UTU v UTM**	0.65501	0.7621	0.75718	0.9435	0.41	0.1065
**UTM v UTL**	0.99915	0 ^1^	0.91642	0 ^1^	0 ^1^	0 ^1^
**UTU v UTL**	0.4216	0 ^1^	0.18507	0 ^1^	0 ^1^	0 ^1^
**TU v TM**	0.9571	0.3333	0.99541	0.7022	0 ^1^	0 ^1^
**TM v TL**	0.9971	0.6757	1	0.2472	0 ^1^	0 ^1^
**TU v TL**	0.99901	0.1652	0.99277	0.1208	0 ^1^	0 ^1^

^1^ indicates *p* ≤ 0.05 (statistical significance).

## Data Availability

The OTU sequences have been deposited in the NCBI Sequence Read Archive under Bioproject PRJNA938155.
